# Autobiographical Memory Content and Recollection Frequency: Public Release of Quantitative Datasets and Representative Classification Analysis

**DOI:** 10.5334/joc.105

**Published:** 2020-06-17

**Authors:** Robert S. Gardner, Hannah S. Anderson, Matteo Mainetti, Giorgio A. Ascoli

**Affiliations:** 1 Center for Neural Informatics, Structures, and Plasticity, Krasnow Institute for Advanced Study, George Mason University, Fairfax, VA, US; 2 Department of Biology, Syracuse University, Syracuse, NY, US; 3 Carnegie Mellon University, Pittsburgh, PA, US; 4 Neuroscience Program and Bioengineering Department, George Mason University, Fairfax, VA, US

**Keywords:** Episodic memory, autobiographical memory content, prospective memory, experience sampling, word-cue technique, human cognition

## Abstract

Autobiographical memory (AM), the recollection of personally-experienced events, has several adaptive functions and has been studied across numerous dimensions. We previously introduced two methods to quantify across the life span AM content (the amount and types of retrieved details) and the everyday occurrence of its recollection. The CRAM (cue-recalled autobiographical memory) test used naturalistic word prompts to elicit AMs. Subjects dated the memories to life periods and reported the numbers of details recalled across eight features (e.g., spatial detail, temporal detail, people, and emotions). In separate subjects, an experience sampling method quantified in everyday settings the frequency of AM retrieval and of mental representation of future personal events or actions (termed prospective memory: PM); these data permit evaluation of the temporal orientation of episodic recollection. We describe these datasets now publicly released in open access (CRAM: doi.org/10.6084/m9.figshare.10246958; AM-PM experience-sampling: doi.org/10.6084/m9.figshare.10246940). We also present examples of data mining, using cluster analyses of CRAM (14,242 AMs scored for content from 4,244 subjects). Analysis of raw feature scores yielded three AM clusters separated by total recalled content. Normalizing for total content revealed three classes of AM based on the relative contributions of each feature: AMs containing a relatively large number of details related to people, AMs containing a high degree of spatial information, and AMs with details equally distributed across features. Differences in subject age, memory age, and total content were detected across feature clusters. These findings highlight the value in additional mining of these datasets to further our understanding of autobiographical recollection.

## Introduction

Autobiographical memory (AM), recollection of the subjective experience of life events, represents an important component of human cognition. AM is thought to have several functions in everyday life (Pillemer, 1992; Bluck et al., 2005; Bluck & Alea, 2011; Klein, 2013; Szpunar et al., 2013) and numerous features of AM retrieval have been widely studied (e.g., Conway & Pleydell-Pearce, 2000; Wagenaar, 1986; Berntsen & Rubin, 2012).

Most relevant to this Data Report, convergent findings suggest that both the content contained in AM (the details retrieved during recollection) and AM retrieval frequency are essential aspects of memory and may interact to support recollection. For example, frequent AM rehearsal enhances content retrieval and phenomenological characteristics associated with memory (Nadel et al., 2007; Svoboda & Levine, 2009; Walker et al., 2009), which in turn modulate source monitoring abilities (Johnson & Raye, 1981; Johnson et al., 1988; Hashtroudi et al., 1990). Moreover, advanced age is associated with both a decrease in the number of AMs generated from experimental prompts (Schlagman et al., 2009) and various changes to the types of content retrieved (Hashtroudi et al., 1990; Levine et al., 2002; Gardner et al., 2015); notably, age-related memory loss of certain details can be attenuated by AM rehearsal (Cohen, 1998).

The recall frequency of prospective memory (PM), broadly defined here as recollection of to-be-experienced events, or future-oriented thought related to potential actions (see Gardner & Ascoli, 2015; D’Argembeau & Van der Linden, 2004), also appears to be an important aspect of cognition (D’Argembeau et al., 2011; Anderson & McDaniel, 2018). For example, mental simulation of future episodes or PM rehearsal can enhance follow-through of planned actions in younger and older individuals (Altgassen et al., 2014; Kvavilashvili & Fisher, 2007). Frequency data of both AM and PM also hold promise to address questions on the temporality of episodic recollection and its function. It was proposed that an episodic memory system is forward-looking (Klein, 2013; Szpunar et al., 2013), to simulate and evaluate future scenarios and inform decision making. Such a framework asserts that a substantial portion of episodic thought is placed in a future context and emphasizes the need for naturalistic measurement of both AM and PM occurrence.

We previously introduced two methods to independently measure across the life span AM content (Gardner et al., 2012; Gardner et al., 2015) and the naturalistic retrieval frequency of AM and PM (Gardner & Ascoli, 2015). The CRAM (cue-recalled autobiographical memory) test (Gardner et al., 2012, 2015) prompted AMs using naturalistic word cues. Subjects dated the memories to life periods and subsequently reported the numbers of details retrieved across eight content categories or features. Previous analysis of the CRAM dataset demonstrated that certain features (i.e., spatial details, people, and objects) contributed more to AM content than others, and that the total content reported in AM modestly increased with the age of the subject, and decreased with the age of the memory (Gardner et al., 2012, 2015).

In separate experiments, experience sampling was used to quantify the frequency of AM and PM in everyday settings (see Gardner et al., 2012; Gardner & Ascoli, 2015). Subjects received random cell-phone prompts throughout daily life and recorded whether they were engaged in AM or PM at the time of each prompt. Thus, the AM or PM hit rate for a given subject reflected the proportion of time they engaged in these types of recollection. Notably, Gardner and Ascoli (2015) found that all ages spent a significant proportion of time (~10%) engaged in AM. However, whereas younger subjects experienced PM equally as often as they did AM, older adults engaged in PM twice as frequently. Together, these findings suggest both past- and future-oriented episodic thought constitute a substantial fraction of cognition, and reveal an age-associated shift in the temporality of episodic recollection.

We have now posted the complete CRAM and AM-PM experience-sampling datasets and related software tools in open access repositories for free public use and further re-analysis. The CRAM dataset can be accessed at doi.org/10.6084/m9.figshare.10246958. The CRAM test interface can be accessed at doi.org/10.6084/m9.figshare.10246949. The experience sampling dataset of AM and PM is available at doi.org/10.6084/m9.figshare.10246940. Here we describe these newly released datasets in detail and report novel cluster analyses of memory content (Anderson & Ascoli, 2019) to illustrate the potential utility of further mining of these data to facilitate a better understanding of AM and PM retrieval.

## Methods

### CRAM

The CRAM (cue-recalled autobiographical memory) test was administered in Excel and web-browser formats and adapted for online use (see Gardner et al., 2012, 2015). Data from subjects were collected either in person under experimenter supervision or online unsupervised. Subjects included both unsolicited internet-browsing individuals (CRAM was indexed by popular search engines between 2013 and 2018) and those recruited from George Mason University staff, faculty, and students and the local community. The test used naturalistic word cues to prompt AMs, which were dated to life periods. The test subsequently collected counts of retrieved content (details recalled) in eight content categories (or features). It was administered in several parts outlined below. For complete detail and full scripts, see Gardner et al., 2012 and doi.org/10.6084/m9.figshare.10246949.

Subjects provided age and demographic information and were then presented with the definition of AM.

“Autobiographical memories are recollections of past episodes directly experienced by the subject. These memories should be of a brief, self-consistent episode of your life. An episode can be as short as a single snapshot and up to a few seconds long.… If the memory you think of refers to a typical and repeated episode that happened regularly or multiple times in your life, you can use it only if you can fixate on a specific individual event. If you can only recall the generic (repeated) event, look for another memory.”

Subjects were subsequently presented with a list of seven word cues and asked to identify (and label) the first AM that came to mind. The word cues were selected randomly from the British National Corpus (natcorp.ox.ac.uk), a collection of written and spoken works. Therefore, the presented word cues should closely match word prompts observed in natural conditions (e.g., during reading or conversation; see Gardner et al., 2012 for processing details of the cue-words).

After AMs were cued, subjects were asked to date each memory to a life period. Each person was presented with temporal bins that divided their life span into ten equal intervals. Subjects could select up to three temporal bins for any given memory.

Participants were subsequently asked to report the number of details recalled in each of eight feature categories: *Things* (objects), *Feelings* (emotional details), *People* (unique individuals), *Places* (spatial details), *Times* (temporal details), *Episodes* (temporally linked events), *Contexts* (other contextual details), and other *Details* (all remaining content, including actions). Feature definitions and presented examples can be accessed at doi.org/10.6084/m9.figshare.10246949 and were previously reported (Gardner et al., 2012).

Feature content was defined as the total number of details reported within a given feature. Total content was defined as the total summed details reported across all features.

For subjects who took the test under experimental supervision, 30 AMs were cued, of which the first two were excluded from analysis. The remaining 28 were dated to life periods, and ten were scored for content. For those who took the test online, an option of four test types was provided. The Atomic test cued, dated, and scored one AM. The Mini test cued, dated, and scored 5 AMs. After completion, the Mini test could be extended (Extended test) with 15 additional AMs cued and dated, of which 5 were scored. The Full test cued 20 AMs, all of which were dated. Of these cued and dated AMs, ten were scored for content (see Gardner et al., 2015 for full detail of test types).

The raw dataset is supplied as Excel and comma separated values files and structured with rows corresponding to individual AMs (doi.org/10.6084/m9.figshare.10246958). Columns link subject demographic information with AM temporal bins and content measures. Information supplied in the dataset for each AM includes a subject ID, subject age (in years), gender, whether English is a native language, if the test was completed in person or online, the test type utilized (Atomic, Mini, Extended, Full), the order in which the memory was cued (memory ID), the life period (temporal bin) to which the memory was dated, and the number of remembered details reported for each of the 8 feature categories. AMs dated to multiple temporal bins are represented in the dataset in multiple rows (one for each associated temporal bin).

### Data Screening

For all analyses reported here, only AMs scored for content across all features were included. Additionally, data were screened for erroneous or otherwise inaccurate or false entries (see Gardner et al., 2015). For example, AMs were excluded from analysis if identical content values were reported for all eight features or features scores were identical across most AMs from a given subject. AMs were also excluded if they contained more than 150 details (mean total content plus three times the standard deviation).

### Cluster analysis of AM content

Screened data (12,654 scored AMs, from 4,060 subjects) were exported to SPSS (IBM) for cluster analyses (all default SPSS parameters selected). Two-step cluster analysis was run to evaluate unique classes of AMs based on raw content scores across features. A k-means cluster algorithm (k = 3) was applied to feature proportions (feature content normalized to total content). A series of ANOVAs was run to assess differences across clusters in total content, the age of the participant, and the age of the memory. In cases where AMs were dated to multiple temporal bins, the mean bin for that memory was computed and used for analysis. Bonferroni correction was applied to multiple comparisons. Statistical significance was interpreted using the criterion of p < 0.05. Data were collapsed across test types with memory as the unit of analysis.

### Within-subject and between-subject content variability

Subjects who scored at least two AMs (10,001 scored AMs from 1,407 subjects), were included in analyses. Unique user IDs were assumed to be from unique individuals. The standard deviation of total content within each subject was computed and its distribution plotted. AMs were then shuffled and randomly re-assigned to each individual and differences in standard deviations across the shuffled and original datasets were assessed using an independent samples two-tailed *t*-test.

### Experience sampling of AM and PM

Cell-phone prompts were used to sample the momentary occurrence in everyday settings of AM and PM. One-hundred and six subjects (18–75 years old) were enrolled from George Mason University and the local community. During an orientation, subjects were provided with definitions of AM and PM. AM was defined as the recollection of an episode from the personal past specific to a particular time and place. PM was defined as the recollection of a task or event that is to occur in the personal future, for example, bringing to mind an intention to stop at the grocery store on the way home from work. PM could also include first-person-perspective thinking of future actions. Thus, our definition of PM overlaps with future-oriented episodic thought (D’Argembeau & Van der Linden, 2004) or mental simulation of future experiences, and differs somewhat from characterizations of PM as successful execution of planned actions or as recollection of previously formed intentions to act (Kvavilashvili & Fisher, 2007; see McDaniel & Einstein, 2007). Full enrollment detail with orientation scripts was previously reported (Gardner & Ascoli, 2015).

Participant cell phone numbers were placed into a custom automatic dialer which initiated random calls. The approximate number of calls and daily calling windows were selected by the individual participant to facilitate call reception; however, when precisely daily calls were made within those temporal windows was random. Subjects were instructed to document the concurrence of calls (prompts) with either an AM or PM. If a call interrupted an AM or PM, the subject marked the type of memory (AM or PM) and estimated the duration (in seconds) of the thought up to the point of the prompt. If the prompt did not interrupt a memory, the subject indicated that the call was noted but no memory was interrupted.

Altogether, subjects received an average of 219 calls over the course of 19 days. Hit rates of AM and PM occurrence were used to compute the proportion of time individuals of various ages engaged in AM or PM. This dataset is publicly supplied for data mining as Excel and comma separated values files and structured with rows corresponding to individual subjects and call (prompt) logs (doi.org/10.6084/m9.figshare.10246940). The columns indicate participant information, the total number of calls received within and across days, and the dates of participation for each subject; subsequent columns link call number with each documented AM or PM and the memory’s estimated duration up to the point of the call. All studies were approved by the George Mason University Human Subject Review Board. Informed consent was obtained prior to participation.

## Results and Discussion

**Figure 1:**
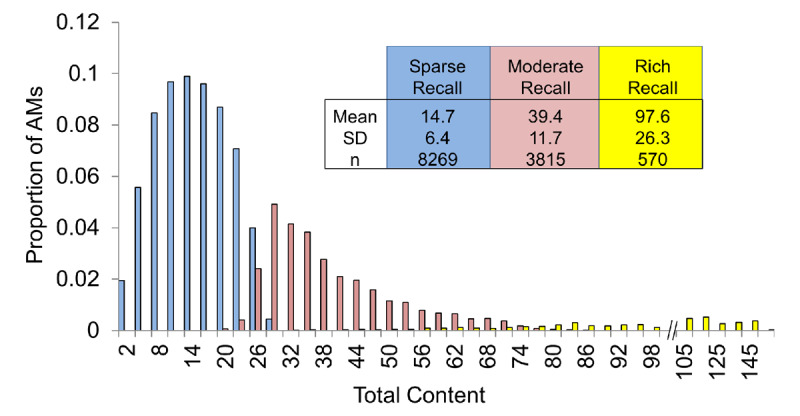
*Cluster analysis of feature counts distinguishes between AMs based on the total amount of detail they contain*. Two-step unsupervised cluster analysis of feature counts identified three distinct classes of AMs: 1) those associated with relatively little content (sparse recall), 2) those with a moderate amount of content (moderate recall), and 3) those relatively rich with content (rich recall). Histograms highlight the distribution of total content scores for AMs within each cluster.

Cluster analyses were applied to the CRAM dataset to identify distinct groups of AMs based on the content they contain. Unsupervised two-step cluster analysis of feature counts produced three clusters of AMs which significantly differed in terms of total content (Figure 1). Specifically, 65.3% (8,269 in total) of the memories were characterized by relatively sparse recall (mean total content = 14.7; SD = 6.4), 30.2% (3,815 in total) by moderate recall (mean total content = 39.4; SD = 11.7), and 4.5% (570 in total) by rich recall (mean total content = 97.6; SD = 26.3). Content for each feature increased from sparse to moderate to rich content clusters (data not shown). Moreover, while the effect sizes were mild, the moderate and rich clusters were associated with significantly older individuals (mean age of subject ± SD = 34.2 yo ± 14.0 and 35.2 ± 13.4 in moderate and rich content clusters, respectively, vs. 32.7 ± 14.0 in the content sparse cluster; p < 0.05). Memory age (mean temporal bin) was equivalent across groups (p > 0.10).

**Figure 2:**
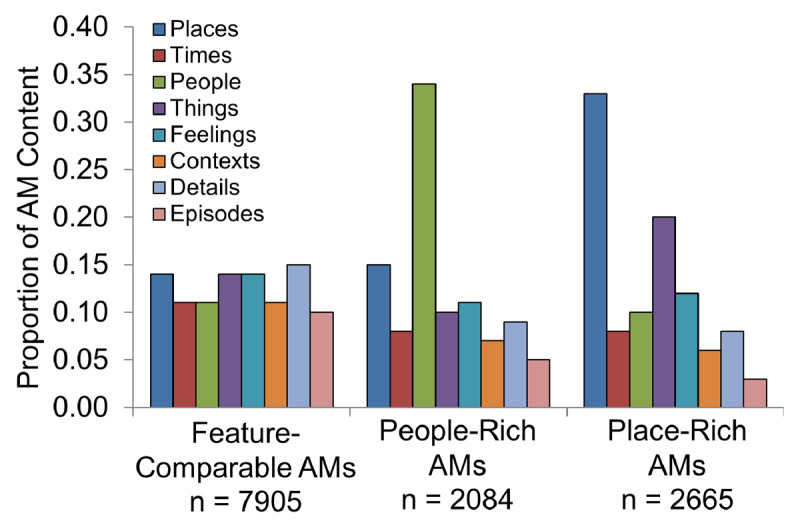
*Analysis of feature contributions to total content reveals unique clusters*. A k-means clustering algorithm (k = 3) produced groups characterized by AMs with comparable feature composition, People-Rich AMs, and Place-Rich AMs.

Further analysis (k-means clustering; k = 3) normalized feature scores to total content to facilitate detection of unique AM groups based on relative feature distributions (Figure 2). Using this approach, memory clusters should stem from distinct feature contributions to total content rather than from differences in absolute feature counts. Cluster 1 (AMs = 7,905) comprised AMs with content similarly distributed across features, i.e., all features contributed similarly (~10–15%) to total content (“feature-comparable” AMs). Memories in Cluster 2 (AMs = 2,084) contained a relatively large proportion of details related to the feature People (~34% of total content) whereas AMs in Cluster 3 (AMs = 2665) contained a relatively large proportion of details related to Places (~33% of total content; with a modestly enhanced contribution from the feature Things). These findings suggest that People and Places (spatial information) may represent robust and/or relatively independent features of recall.

Total content among feature-comparable AMs (mean = 29.9 details, SD = 22.2) was significantly greater than that among the other two feature clusters (p < 0.05), with AMs richly represented by People (20.7 details, SD = 19.1) showing modestly higher content scores than those represented by Places (17.9 details, SD = 18.5; p < 0.05). Furthermore, People-rich AMs were associated with slightly younger individuals (mean age = 31.8 years old, SD = 14.2) than were feature-comparable AMs (mean age = 33.6 years old, SD = 14.0; p < 0.05) or Place-rich AMs (mean age = 33.2, SD = 13.7; p < 0.05). Finally, Place AMs were somewhat more remote (mean temporal bin = 4.6, SD = 2.8) than were feature-comparable AMs (mean temporal bin = 5.0, SD = 2.8; p < 0.05) or People AMs (mean temporal bin = 5.1, SD = 2.6; p < 0.05).

Although cluster effects on the age of the memory and individual were significant and consistent with prior reports, for example, of increased content or ratings of recollection among older individuals (Gardner et al., 2015; Janssen et al., 2011; however, also see Levine et al., 2002), the magnitudes of these differences were mild; these effects appear to be driven by the relatively large sample size of AMs in the dataset and should be interpreted accordingly. Nevertheless, these analyses showcase the power of this AM dataset to flesh out small content differences across conditions.

**Figure 3:**
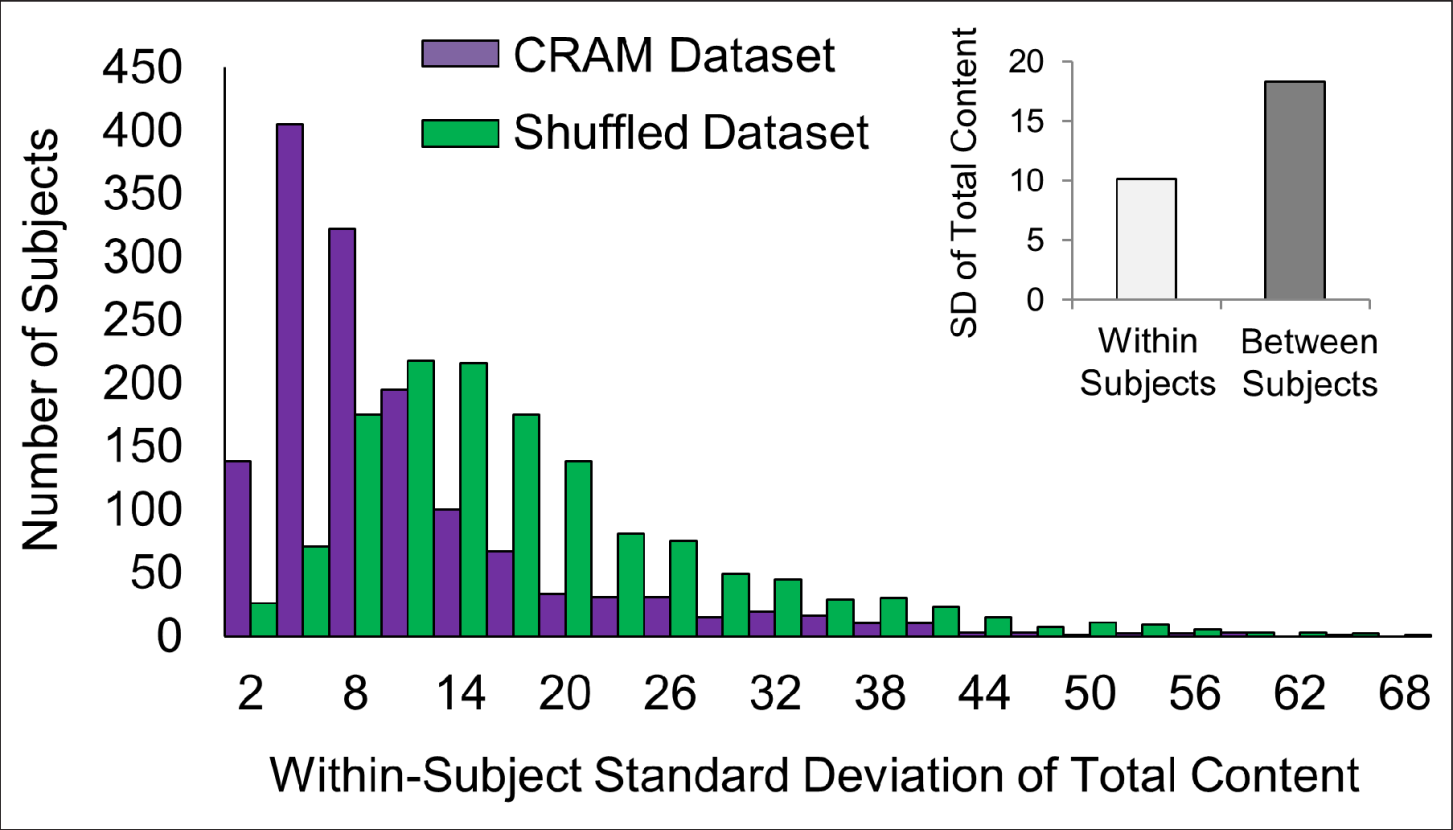
*Within-subject variability in total reported content is less than that between subjects*. Between-subject standard deviation was almost twice as large as that within-subjects (inset). To ensure the relatively low within-subject variability across AM content was not due to chance, AMs were shuffled and then randomly reassigned to individual subjects. Lower within-subject AM content variability was found in the original dataset compared with that in the shuffled dataset (mean SD = 10.1 vs. 17.7, respectively; p < 0.05). Histograms of the two datasets are presented. Number of subjects = 1,407; Number of AMs = 10,001.

To highlight the value of these data for examining individual differences in recollection, we also contrasted within- and between-subject variability in AM content (Figure 3). Restricting analysis to subjects who scored at least two AMs, we found that the between-subject standard deviation (SD = 18.3), was almost twice as large as that within subjects (mean SD = 10.1), suggesting that, on average, AM content varies relatively more from person to person, than it does between AMs from the same person.

To evaluate statistically if the relatively low within-subject variability in AM content was due to chance, AMs were shuffled and randomly re-assigned to individuals with the number of AMs reported from a given subject kept constant. Indeed, lower measures of within-subject content variability were found in the original dataset compared to that in the shuffled dataset (mean SD = 10.1 vs. 17.7, respectively; p < 0.05; Figure 3), corroborating our initial findings.

In conclusion, this report describes two experimental methods and resulting datasets, now publicly available for data mining, that measure the content (amount of types of details) retrieved in AM as well as AM and PM retrieval frequency in natural settings. We stress that our methods rely on subjective accounts of recall; for example, CRAM relies on participant counts of the details contained in memory assigned to feature categories and contrasts with studies using experimenter-scored participant narratives to quantify recalled features or lab-created events that can assess the veridicality of recollection (e.g., Bartlett, 1932; Hashtroudi et al., 1990; Levine et al., 2002; see Gardner et al., 2012, 2015 for detailed comparisons of the CRAM method to alternate approaches). Nonetheless, previous reports showed that AM data collected with the CRAM test reproduce several robust features of memory suggesting it is a valid tool to cue AMs and collect content measures (Gardner et al., 2012, 2015). For example, AMs scored through CRAM demonstrate a temporal decay in content consistent with that previously reported (e.g., Levine et al., 2002; Janssen et al., 2011); AMs elicited with CRAM also yield classic features related to their life span distribution: a retention interval, reminiscence bump, and childhood amnesia (Janssen et al., 2011; Rubin and Schulkind, 1997). Frequency measures of recollection estimated from our experience sampling technique are also in line with more recent reports using similar procedures (e.g., Anderson & McDaniel, 2018).

Our methods did not distinguish between voluntary/effortful recall and involuntary/spontaneous recall (Berntsen & Rubin, 2012). Interestingly, prior work suggests that the method used to elicit memories alters the predominant mode of retrieval (e.g., Barzykowski et al., 2019), which may relate to changes in recalled content and retrieval frequency (see also Berntsen 1998; Schlagman & Kvavilashvili, 2008). Future work to characterize the typical retrieval mode of memories sampled by our studies should augment our understanding of the presented analyses and facilitate future data mining of these datasets.

Overall, the selected characterizations of AM content reported here (cluster analyses and variability measures) are examples to highlight just a few of the many ways in which these rich datasets may be used to further probe the content and frequency of episodic recollection.

## Data Accessibility Statement

All datasets are deposited in open access repositories and are freely available for public use. The complete CRAM (cue-recalled autobiographical memory) dataset can be accessed at doi.org/10.6084/m9.figshare.10246958. The CRAM test interface can be accessed at doi.org/10.6084/m9.figshare.10246949. The complete experience sampling dataset of autobiographical and prospective recollection can be accessed at doi.org/10.6084/m9.figshare.10246940.
